# Plasmonic signal modulation at sub-GHz frequency via on-chip integration of tunnel junctions

**DOI:** 10.1515/nanoph-2023-0720

**Published:** 2024-01-03

**Authors:** Fangwei Wang, Baohu Huang, Yan Liu, Siping Gao, Yongxin Guo, Qian Zhang

**Affiliations:** National University of Singapore, Singapore, Singapore; National University of Singapore (Chongqing) Research Institute, Chongqing 401123, China; School of Chemistry and Chemical Engineering, Chongqing University, Chongqing 400044, China

**Keywords:** quantum tunneling, plasmonic signal, light emission, RF frequency, junction breakdown

## Abstract

Plasmonic technology offers one of the most promising solutions to achieve on-chip integration of nanoscale and fast modulation circuits using surface plasmon polaritons (SPPs) as the information carriers. However, the potential of modulation speed of plasmonic signals has not been fully tapped. In this paper, we have demonstrated the plasmonic signal can be modulated at the bandwidth of sub-GHz (>100 MHz) via the on-chip integration of tunnel junctions. We also find that the lifetime of tunnel junctions under AC conditions can be improved significantly compared with the DC counterparts, which allows us to investigate and visualize the real-time breakdown process of tunnel junctions. Our implementation of plasmonic signal modulation at sub-GHz frequency paves the way toward potential industrial applications of on-chip plasmonic circuits.

## 1 Introduction

CMOS microelectronics has followed the footprint guided by the famous Moore’s law in the past 5–6 decades, where the integrated density of transistors doubles every 18 months. However, heat dissipation and RC (resistor-capacitance) delay of electronic interconnects hinder the modulation speed of electronic signals well below 10 GHz. Silicon photonics provides a new path towards ultra-large bandwidths and fast modulation speeds (∼THz) since the signals are transmitted via optical waveguides, which, unfortunately, are typically bulky and not easy to integrate with CMOS electronics. This dilemma of size mismatch between nano-footprint electronics and diffraction-limited photonics complicates further integration to realize nano-optoelectronic circuitry [1], [2], [3]. Plasmonic technology has been regarded as one of the most promising solutions to achieve on-chip integration of nanoscale and fast modulation circuits using surface plasmon polaritons as the information carriers. To make all the components on the same chip, it is needed to encode, transmit, and read out electrical signals via surface plasmon polaritons [4], [5], [6]. Recently, intense research efforts have been focused on the electrically-driven plasmonic components based on metal-insulator-metal tunnel junctions for on-chip application [4], [5], [7], [8], [9], [10]. The main appeals over tunnel junctions lie in the ultrasmall footprint (atomic-scale resolution for scanning tunneling microscope, STM) and potential ultrafast modulation speed (∼100 THz) [10], [11], [12]. The scale shrinking of metal–insulator–metal (MIM) tunnel junctions from microscale to nanoscale is essential for on-chip integrated circuits. In principle, the modulation speed of tunnel junctions is only limited by the tunneling timescale at the order of a few femtoseconds [13], [14]. However, plasmonic components based on tunnel junctions are often driven by direct currents, which hinder the further development of high-frequency transmission of plasmonic signals. In other words, it is urgent and necessary to generate, propagate, modulate, and detect plasmonic signals at AC conditions with a high driving frequency. To better benchmark the state of the art of plasmonic components based on AC-driven tunnel junctions, we have summarized and compared some vital parameters of electrical performance reported in previous literature (Table S1) [4], [5], [7], [10], [15]. Despite this significant progress, there are still plenty of challenges that need to be resolved for industrial applications. Firstly, the potential of direct modulation speed of electronic-plasmonic signals has not been fully investigated and the modulation frequency was demonstrated as high as 1.0 MHz [5], [7]. Secondly, the AC (alternating current) frequency-dependent light emission from MIM junctions was not studied systematically). Lastly, the long-term stability and breakdown mechanism were often ignored [16], [17]. It is worth mentioning that the electrical breakdown process of tunnel junctions is not trivial to explore or visualize in real time since the breakdown timescale can be quite fast [17], [18].

Here in this paper, we have demonstrated the plasmonic signal can be modulated at the bandwidth >100 MHz via the on-chip integration of two MIM junctions, which can be pushed to an even higher frequency by overcoming the equipment limitation and impedance mismatch of electrical connections. We also show that the light emission due to quantum tunneling can be modulated and detected at a frequency of 100 MHz limited by the impedance mismatch and the efficiency of the optical setup. Typically, plasmonic components based on tunnel junctions are driven under direct-current (DC) conditions, and the electrical breakdown mechanism may be attributed to the electromigration of metallic ions or local heating at metal-oxide interfaces [17], [18]. In this report, we demonstrated that the electrical breakdown process of tunnel junctions can be slowed down effectively and visualized via optical microscopy in real time under AC-driven conditions. Our result provides a new way to explore the breakdown mechanism of tunnel junctions in real-time, for example, we may use TEM (transmission electron microscopy) or STM technique to visualize the atomic movement since the breakdown process is slowed down to a few seconds.

## 2 Result and discussion

**Figure 1:**
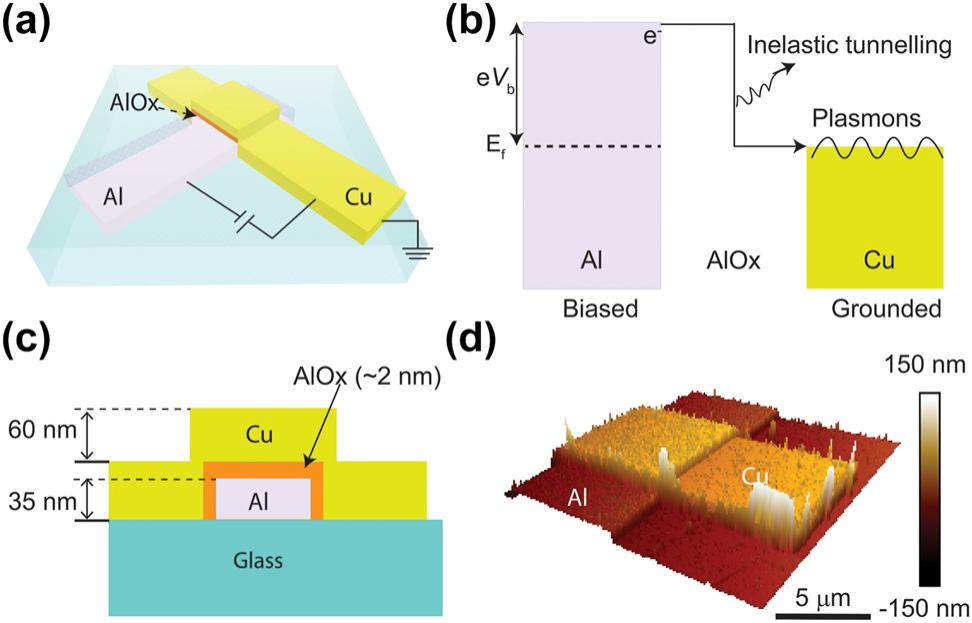
Plasmonic exciatation via quantum mechanical tunneling. (a) Device schematic of Al–AlO_X_–Cu tunnel junctions. (b) SPP excitation mechanism via IET. (c) Cross-section profile along the Cu waveguide. (d) Three-dimensional AFM image in perspective.

**Figure 2:**
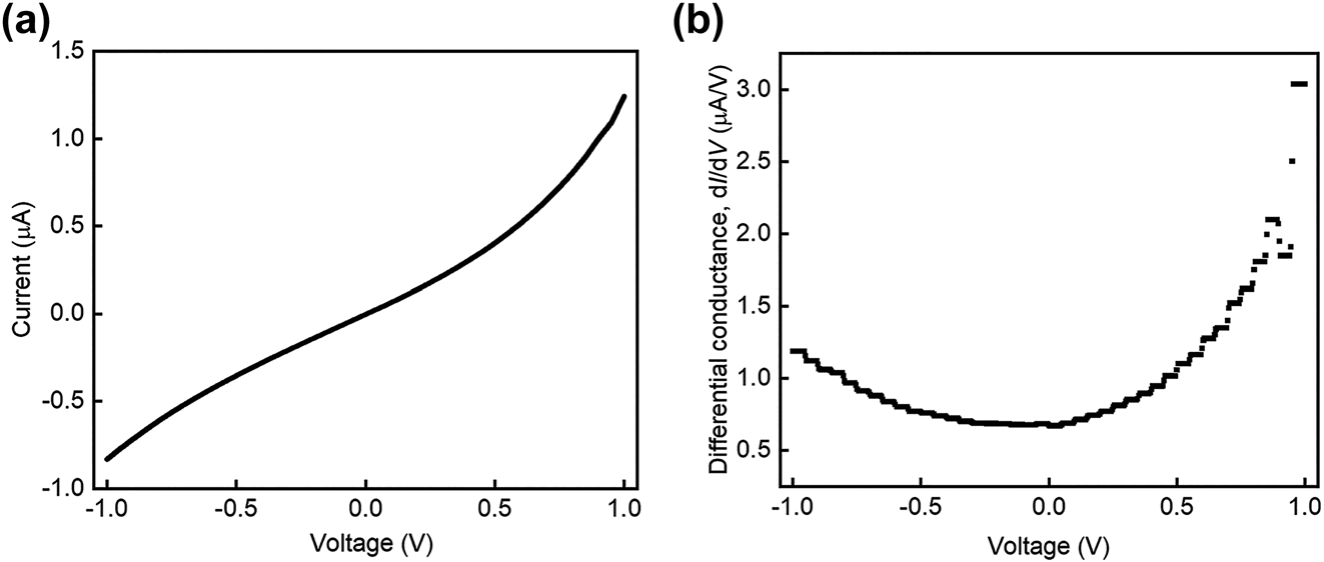
*I*(*V*) characteristics of tunnel junctions. (a) *I*(*V*) curve and (b) differential conductance of tunnel junctions measured at 300 K. Both curves were averaged over five cycles sweeping from −1.0 V to 1.0 V.

Figure 1a illustrates the conceptual schematic of Al–AlO_X_–Cu tunnel junctions, comprised of bottom Al electrodes, top Cu electrodes, and native-grown AlO_X_ assembly on borosilicate coverslips. Native-grown AlO_X_ serves as a stable and high-quality tunnel barrier due to the self-limiting growth process and its large bandgap (∼7.0 eV). The tunneling current mostly comes from elastic tunneling, where the electronic energy is maintained before and after quantum tunneling. A small proportion of electrons can also tunnel inelastically with the lost energy quanta coupled to phonons, photons, and other optical modes. Due to the ultra-large optical local density of states of MIM-SPP cavity mode, the inelastic electron tunneling (IET) rate can exceed 10 % [10], [11]. Figure 1b illustrates the excitation mechanism based on IET, where the lost energy quanta couples to all the available SPP modes. Figure 1c shows the cross-section profile of tunnel junctions, where the Al (35 nm) and Cu (60 nm) electrodes sandwich an AlO_X_ layer (∼2 nm). To visualize the propagating property of SPP modes, a plasmonic Cu waveguide with a length of 5 μm was connected to tunnel junctions of 5 × 5 μm^2^ (Figure 1d). To verify the tunneling behavior of fabricated devices, we have conducted the electrical performance of tunnel junctions. Figure 2a and b shows a typical nonlinear *I*(*V*) response and its associated parabolic differential conductance (d*I*/d*V*), indicating coherent quantum mechanical tunneling of the electrons. To ensure that quantum tunneling dominates the charge transport mechanism, we also demonstrated the tunnel current is almost independent of temperature as expected for coherent tunneling in our previous report [4]. We acknowledge that the potential oxidation of Cu remains one of the major challenges of Cu as a cheap and high-quality plasmonic material. Thus, we have shown that the coating of a SiO_2_ cladding layer on top of Al–AlO_X_–Cu junctions is helpful to prevent the oxidation of Cu surfaces in a couple of days in our previous work [7].

**Figure 3:**
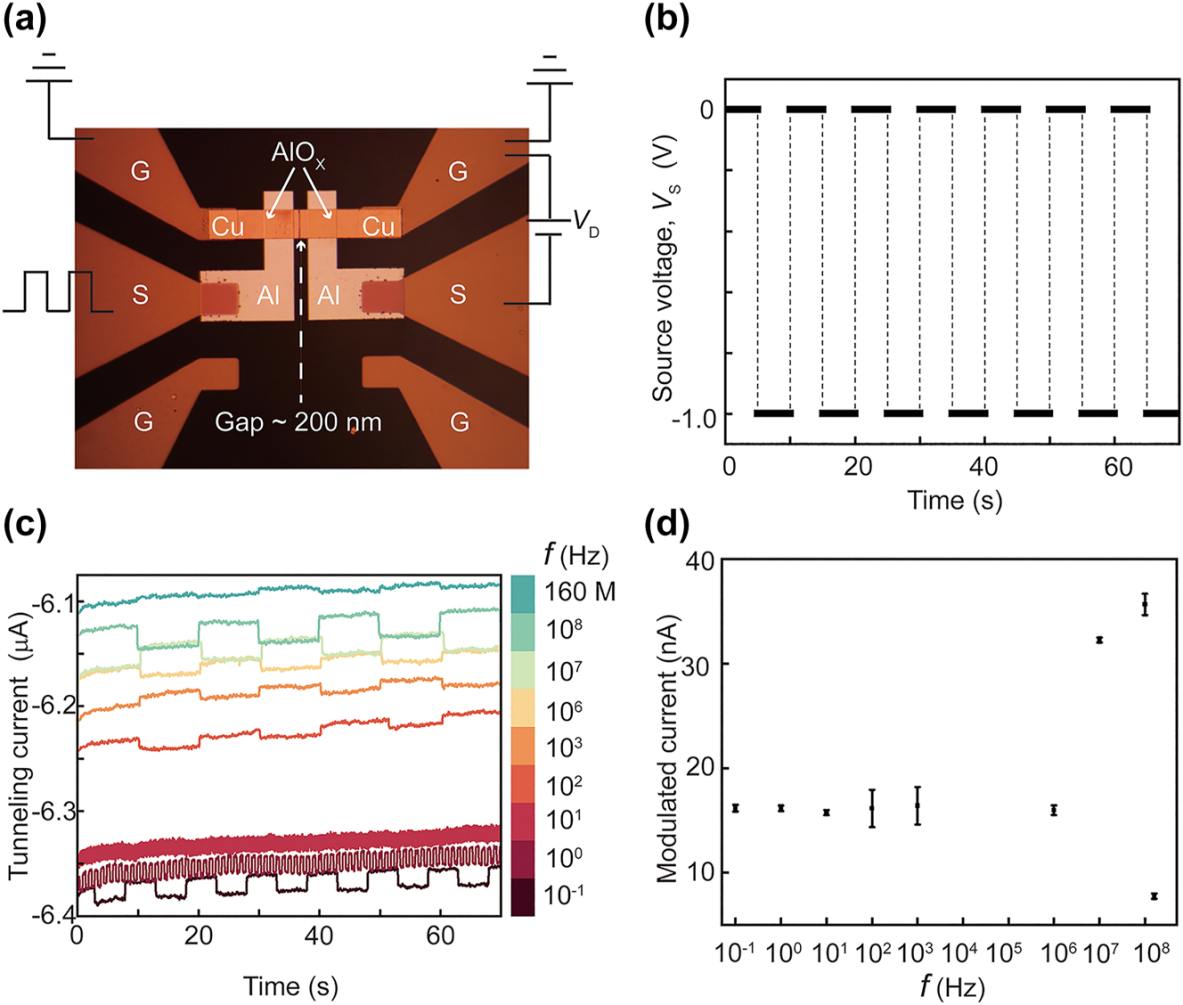
Demonstration of plasmonic signal modulation at sub-GHz frequency via on-chip integration of tunnel junctions. (a) Optical image of the electronic-plasmonic transducer consisting of two Al–AlO_X_–Cu tunnel junctions connected by a Cu waveguide. A square waveform voltage (*V*_S_) was applied on one MIM tunnel junction (source), and the response current (*I*_D_) was recorded with a constant voltage applied to the MIM tunnel junction (detector). GSG pads are designed to minimize the impedance mismatch between the input signals and devices to be measured. (b) An example of time traces of square waveform of source voltage with a period of 10 s. (c) Time traces of current at various modulated frequencies ranging from 0.1 Hz to 160 MHz. (d) Modulated current as a function of applied modulation frequencies.

Figure 3a shows the optical image of designed plasmonic transducers, which consist of two Al–AlO_X_–Cu tunnel junctions as source and detector, respectively. The GSG (ground-source-ground) pads, line resistance, and taper structure were designed specifically to minimize the impedance mismatch between input electrical signals and the tunnel junction. The SPP source was applied with square waveform signals by the function generator at various frequencies ranging from 0.1 Hz to 160 MHz (the available maximum frequency of the function generator is 160 MHz). In our previous report, we provided a detailed analysis of SPP modes at the junction area and also at the end of Cu waveguides at DC conditions [4]. To study the role of applied AC frequency on the excited and propagated SPP modes, we have collected real-plane and back focal-plane images at the junction area and the waveguiding edge. Supplementary Section S2 concludes that the driving AC frequency does not alter the excited and propagated SPP modes but only modulates the light-emitting intensity. The SPP detector was connected with the Keithley 6430 source meter to measure the time trace of the response current simultaneously biased at constant voltages. Since the response time of Keithley is limited to a few ms, we have turned on and off the function generator for a period of 10 s to visualize the modulation when the applied frequency is above 100 Hz. Taking a frequency of 0.1 Hz as an example, Figure 3b shows that the applied source voltage ranges from 0 to a fixed negative bias (−1.0 V) at the interval of 10 s.

Tunnel junction can not only act as a plasmonic source but also function as an on-chip plasmonic detector. The relevant SPP detection mechanism however is still under debate due to the complex interplay between quantum charge transport and nano optics [5], [19]. One possible explanation could be based on the (photon) plasmon-assisted tunneling (PAT) process, where an oscillating field is induced as plasmonic signals reach the detection junction interfaces [5], [20]. Thus, the tunneling current can be modulated since the oscillating field can alter the tunneling barrier heights. It should be noted that the electronic-plasmonic modulators are connected with a Cu plasmonic waveguide integrated with a designed 200 nm width gap, which only allows SPP propagation and prevents electrons from transmission. By using the same structure, Du and co-workers have demonstrated the significant role of gap size on SPP detection efficiency to exclude any other electrical parasitic effects (capacitive or inductive coupling) [5]. Figure 3c presents the recorded time traces of the detector current at constant voltages, where modulated voltages were applied to the SPP source at different frequency values. We found that the modulated current at the SPP detector followed the same square waveform, suggesting that electrical signals can be encoded, transmitted, and decoded within the plasmonic circuits.

One of the most appealing advantages of our demonstrated electronic-plasmonic modulators is the potential working frequency is not limited by the lifetime of intermediate excitons as most light-emitting diodes do. The SPP generation and detection mechanisms are based on inelastic tunneling and plasmon-assisted tunneling mechanisms, respectively. Thus the ultimate modulation speed is restrained by the tunneling time of electrons through the barriers at the order of a few femtoseconds [13], [21]. In other words, there should be no obvious frequency dependency in our applied AC frequency ranges (<1.0 GHz). However, experimental electronic-plasmonic modulation could also be limited by the RC time constant of the junction itself as well as the impedance matching of electric connections. Figure 3d plots the modulated current as a function of applied frequency. We find that plasmonic signals with a maximum modulation speed of 160 MHz can still be transmitted and detected using SPPs as information carriers. To better understand the frequency-dependent modulation of plasmonic signals, it is crucial to measure the RC constant of tunnel junctions and extract the corresponding cutoff frequency. Figure S4b illustrates the measured and fitted impedance plotted against the applied RF frequency and the corresponding value is fitted to be 8.5 GHz, which is 1–2 orders of magnitude higher than the applied maximum frequency. Since the impedance between the junction and the input signals is not well matched, there is a dip in the reflection coefficient (from the measured junction impedance) between 10 and 100 MHz (Figure S5), meaning the power delivered from the power source to the junction has a peak within this frequency range. When the applied frequency is close to the cutoff frequency (GHz), tunnel junctions reflect almost all the incident electrical power. This is the reason why the modulated current remains constant when the applied frequency ranges from 0.1 Hz to 1.0 MHz and then increases beyond 10 MHz and finally decreases shapely at 160 MHz. However, we have to emphasize that the maximum RF frequency is not limited by the tunnel junction itself (RC delay) but due to the impedance mismatch between the RF signals and devices. The modulation frequency can be further pushed and challenged to GHz or even above with proper measurement design.

**Figure 4:**
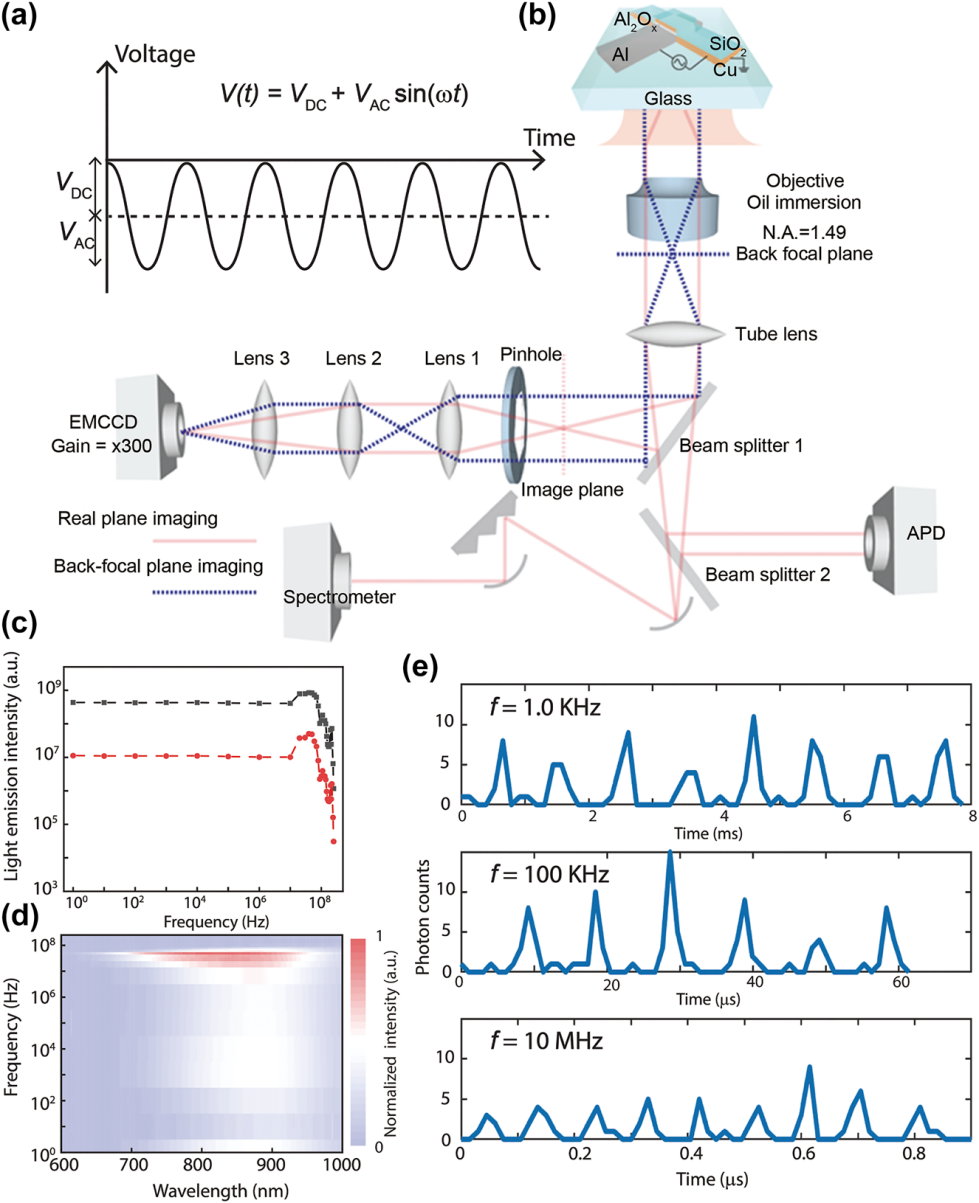
RF modulation of light emission via quantum tunneling. (a) We have set up homemade optical measurement equipment and installed various collection and detection techniques (electron multiplying CCD camera, back focal plane imaging, spectrometer, and avalanche photodiode). (b) Al–AlO_X_–Cu tunnel junctions were biased by an RF signal in the waveform of *V*(*t*) = *V*_DC_ + *V*_AC_ sin(*wt*). (c) Light emission intensity collected via avalanche photodiode as a function of applied RF frequency. (d) Normalized spectral graphs collected at different RF frequencies. (e) Normalized photon counts collected at RF frequencies of 1.0 KHz, 100 KHz, and 10 MHz, respectively.

A common and direct method to detect SPP signals is to collect SPP-scattered light emission due to the local surface roughness. However, it should be noted that light emission collected from biased tunnel junctions not only comes from SPP scattering but photons can also be emitted during the inelastic electron tunneling process [22], [23], [24]. To ensure SPPs were indeed generated, transmitted, and detected within our plasmonic transducers, we have also collected RF-modulated light emission from biased tunnel junctions. SPP signals can only be the information carriers for our plasmonic transducers for two reasons: (1) The emitted photons cannot be transmitted along metallic waveguides. (2) We have designed a gap of 200 nm along the metallic waveguide to isolate the plasmonic source and detector electrically. Figure 4a shows the RF modulation measurement setup for Al–AlO_X_–Cu tunnel junctions, which were biased by a DC-offset AC signal. Figure 4b illustrates the device schematics and electrical connection. Figure 4c and d plot the light emission intensity and spectra as a function of the applied RF frequency. We found that the light-emitting intensity remains constant below 10 MHz, rises significantly until 60 MHz, and declines sharply beyond 100 MHz due to different matching conditions of AC impedance at various RF frequencies. The response of light-emitting intensity as a function of applied frequency is quite similar to the counterpart of plasmonic modulation due to the change of reflection coefficient at various frequencies. We also note that the spectral shape and position do not shift obviously at RF frequency below 10 MHz and then change accordingly at RF frequency higher than 10 MHz. Figure 4e demonstrates the normalized photon counts collected at RF frequencies of 1.0 KHz, 100 KHz, and 10 MHz, respectively. The normalized photon counts remain constant and follow the RF waveform at each applied frequency, indicating that SPPs and photons are indeed excited via the quantum tunneling process at RF input signals.

**Figure 5:**
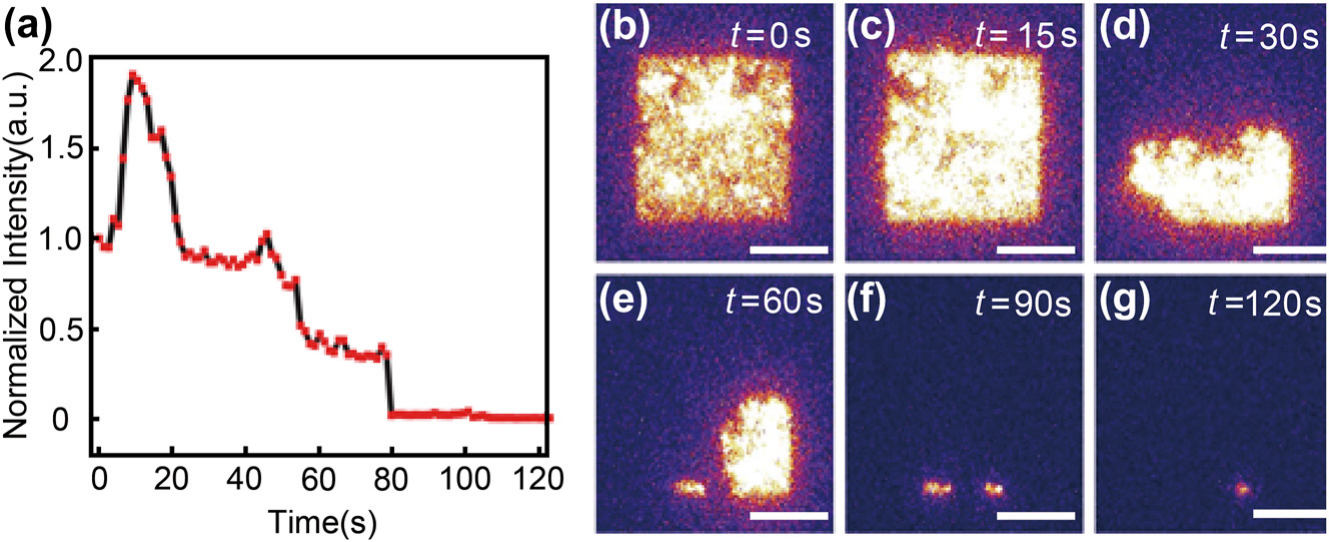
Real-time direct visualization of tunnel junction breakdown. (a) Time-dependent normalized light emission intensity when a sinusoidal waveform voltage sweeps from −3.4 V to + 1.0 V was applied to tunnel junctions. (b–g) Optical images of real-plane light emission at different time intervals. The intensity of all the images was normalized for comparison. The scale bar is 5 μm in length.

One of the most urgent issues hindering the further application of tunnel junctions is the long-term device stability, hence, it is vital to understand how the tunnel junctions break down and cause device failure during operation [16], [25], [26]. Typically, tunnel junctions are biased by DC voltages and the breakdown process is too fast to be captured and visualized in real time. We have demonstrated that the lifetime of tunnel junctions under AC conditions can be improved significantly since the alternating electric field can reduce the electromigration and local heating at oxide-metal interfaces in our previous report [7]. Figure 5 shows the real-time direct visualization of tunnel junction breakdown for the first time when the tunnel junctions were biased using RF signals. To better visualize the light emission of the junction breakdown process, the area of tunnel junctions is designed as 10 × 10 μm^2^. As is shown in Figure 5a, the light-emitting intensity increases sharply and then decays as time goes by. We also note that the light-emitting areas become smaller and smaller and decay to a specific spot and the tunnel junction fails to operate (Figure 5b–g). In our previous report, we also observed the same trend that tunnel junctions will become short circuits and then evolve into open circuits, which is likely caused by local Joule heating or electrical deterioration of insulating layers [7]. It is worthy to clarify the relation between the AC frequency and the long-term stability of plasmonic tunnel junctions. On one hand, if the current remains unidirectional and no reverse voltages are applied, we understand that higher frequency may lead to more intense local heating accumulation, which can fasten the breakdown process of tunnel junctions. On the other hand, the charge accumulation over the metal–insulator interface is significantly reduced at alternating electric fields (voltages are applied at negative and positive biases periodically). Additionally, the bidirectional currents can further minimize electromigration and diffusion of metallic atoms across the metal-insulator interface. Thus, the breakdown process can be slowed down effectively, which enables direct visualization at an order of a few seconds. We also found that the lifetime of plasmonic tunnel junctions shows no trend as a function of applied AC frequencies ranging from 1.0 Hz to 10.0 MHz in our previous report [7]. Our result provides a new way to explore the breakdown mechanism of tunnel junctions in real-time, for example, we may use the TEM or STM techniques to visualize the atomic movement since the breakdown process can be slowed down to a few seconds [27], [28].

## 3 Conclusions

In summary, we have demonstrated that plasmonic signals can be generated, transmitted, and detected via on-chip integration of tunnel junctions and the maximum modulation speed can be as high as 100 MHz. There are plenty of scopes to further push the modulation speed up to a few GHz and above with proper electrical designs to match the impedance of the input signals and the tunnel junctions. We have also shown that RF modulation of light emission via quantum tunneling can operate at a frequency of hundreds of MHz, which further validates that SPPs can act as information carriers. Our result taps the potential application of high-frequency (GHz and above) plasmonic circuits and provides a platform to integrate the plasmonic components on the same chip. Other than that, for the first time, we have captured and visualized the real-time breakdown process of tunnel junctions, which offers a new path to investigate and understand the device failure mechanism since the device breakdown process can be slowed to a few seconds.

## 4 Materials and methods

### 4.1 Device fabrication flow

Our electronic-plasmonic transducers consist of two Al–AlO_X_–Cu tunnel junctions, where one serves as the SPP source and the other acts as the SPP detector. Our devices are fabricated on top of borosilicate coverslips (Paul Marienfeld GmbH, 22 × 22 mm, 0.16–0.19 mm thick), which are transparent at near-infrared and visible wavelength so that light emission can be collected from the back side of the glass substrates. To begin with, the area of contact pads is 100 μm × 100 μm, which is defined by laser writer photolithography and followed by the electron beam deposition of 3 nm Ti and 30 nm Au (The lithography details, including spin coating, type photoresist, and lift-off parameters, are described in our previous work [4], [5], [7]). EBL techniques (JEOL, JBX-6300FS) were utilized to pattern the bottom Al electrodes with a current set at 5 nA and a beam size of 50 nm. After developing in MIBK: IPA (volume ratio = 1:3) solution, a 35 nm Al layer (AJA, 99.99 %) was deposited as the bottom electrode via electron beam deposition. It is worth mentioning that the thickness of native-grown AlO_X_ plays a crucial role in the electrical performance of tunnel junctions. In our work, the thickness of native-grown AlO_X_ is well controlled within the range of 1–3 nm by etching the Al surface and exposing the fresh Al surface at ambient conditions for 30 min. Last but not least, an extra step of EBL was used to pattern the top electrodes, followed by 60 nm Cu sputtering deposition (AJA International, ATC-Orion 8 UHV). Meanwhile, a 180 nm gap was also introduced along the Cu waveguide to disable the propagation of electronic signals, only allowing the transmission of plasmonic signals.

### 4.2 Optical light emission characterization

The electrical characterization was measured via a source meter (Keithley 6430 instruments) and the voltages were applied to the Al electrodes using the homemade LabView program. The light emission from biased tunnel junctions is collected from the back side of glass substrates using an inverted microscope system (Nikon Eclipse Ti-E, oil objective, numerical aperture = 1.49). Different light-emitting signals are collected by respective components via various optical paths (Figure 4a). The light-emitting images and spectra were visualized via an electron-multiplying CCD (EMCCD, iXon Ultra 897) and a spectrometer (Andor, Shamrock 122 303i) with an integration time of 2 min. The AC signal-driven emitted photons are then counted by an avalanche diode. A pinhole structure was introduced along the light path between the beam splitter and the lens to collect the light emission from regions of interest.

### 4.3 Plasmonic signal modulation measurement

The SPP source was applied with square waveform signals by the function generator at various frequencies ranging from 0.1 Hz to 160 MHz (the available maximum frequency of the function generator is 160 MHz). The SPP detector was connected with the Keithley 6430 source meter to measure the time trace of the response current simultaneously biased at constant voltages. Since the response time of Keithley is limited to a few ms, we have turned on and off the function generator for a period of 10 s to visualize the modulation when the applied frequency is above 100 Hz. The GSG RF probes (MPI-T26A, impedance = 50 Ω, maximum frequency up to 26 GHz), RF connection cables (SMM12, impedance = 50 Ω, maximum frequency up to 26 GHz), line resistance (50 Ω), and taper structure were designed specifically to minimize the impedance mismatch between input electrical signals and the tunnel junction.

## Supplementary Material

Supplementary Material Details
