# Supplementary material for: Plasmonic signal modulation at sub-GHz frequency via on-chip integration of tunnel junctions
Source: Nanophotonics. 2024 Jan 3;13(2):209–16. doi: 10.1515/nanoph-2023-0720 (PMC11501128; doi:10.1515/nanoph-2023-0720)
Supplement: Supplementary file 1 — Supplementary Material Details [file j_nanoph-2023-0720_suppl_001.docx]

Supplementary Information

Plasmonic signal modulation at sub-GHz frequency via on-chip integration of tunnel junctions

*Fangwei Wang^1,2^, Baohu Huang^1^, Yan Liu^1^, Siping Gao^1^, Yongxin Guo^1,2*^, Qian Zhang^2,3*^*

^1^Department of Electrical and Computer Engineering, National University of Singapore, Singapore

^2^Intelligent Sensing and AI Center, National University of Singapore (Chongqing) Research Institute, Chongqing 401123, China

^3^School of Chemistry and Chemical Engineering, Chongqing University, Chongqing 400044, China

*Author to whom correspondence should be addressed: [eleguoyx@nus.edu.sg](mailto:eleguoyx@nus.edu.sg) and [qian.zhang@nusricq.cn](mailto:qian.zhang@nusricq.cn)

## S1 Comparison with state-of-the-art plasmonic modulation based on tunnel junctions

## Tunnel junctions have been regarded as one of the most promising platforms to achieve on-chip integrated circuits with ultrafast modulation speed around THz due to the ultrasmall device footprint and femtoseconds quantum tunneling timescale. To better demonstrate the novelty of this work, we have conducted an exhaustive literature review shown in Table S1, and compare our result with the state of the art of plasmonic modulation based on tunnel junctions. We would love to emphasize three major aspects and new insights of our work to separate from the previous reports. To begin with, we have demonstrated electronic-plasmonic signal modulation at sub-GHz frequency for the first time. To our knowledge, the electronic-plasmonic modulation frequency was demonstrated as high as 1 MHz in previous literature.^1^ Our result shows that the modulation frequency can be further pushed and challenged to GHz or even above with proper measurement designs and impedance-matching device configurations. Here in this work, we have utilized GSG configurations to minimize the impedance mismatch between the RF signals and the tunnel junctions. On the contrary, GS (ground-signal) connections are typically used in other reports without considering possible impedance mismatch at high frequencies.^1-4^ In addition, we have replaced all the DC connections (wires and tips) with RF counterparts with a maximum frequency of 3 GHz. Secondly, we have investigated systematically the role of AC driving frequency on the light-emitting spectra and intensity via quantum tunneling. We found that the light-emitting intensity remains constant below 10 MHz, rises significantly until 60 MHz, and declines sharply beyond 100 MHz due to different matching conditions of AC impedance at various RF frequencies. Last but not least, we have paid much attention to the breakdown process of tunnel junctions. One of the most urgent issues hindering the further application of tunnel junctions is the long-term device stability, hence, it is vital to understand how the tunnel junctions break down and cause device failure during operation. Here, we first demonstrate the real-time direct visualization of tunnel junction breakdown when the tunnel junctions were biased using RF signals to slow down the breakdown process.^2^ Our result provides a new way to explore the breakdown mechanism of tunnel junctions in real time.

Table S1 Comparison with state-of-the-art plasmonic devices and the modulation speed measurement based on tunnel junctions

| Device | CMOS | Electron-photon | *f*_max1_ | Electron-plasmon | *f*_max2_ | Breakdown | Ref |
| --- | --- | --- | --- | --- | --- | --- | --- |
| Al-AlOx-Au | × | × | N.A. | √ | 1 MHz | × | 1 |
| Au-hBN-Au | × | √ | 1 GHz | × | N.A. | × | 2 |
| Al-AlOx-Cu | √ | √ | 10 MHz | × | N.A. | √ | 3 |
| InGaN/GaN | √ | √ | 20-60 Hz | × | N.A. | × | 4 |
| Al-AlOx-Cu | √ | √ | 250 MHz | √ | 160 MHz | √ | This work |

## S2. SPP modes analysis at tunnel junction area and the end of Cu waveguides

In our earlier result, we have already presented a detailed analysis of SPP modes generated in Al-AlOx-Cu tunnel junctions at DC conditions.^[5]^ Here we provide an elaborate discussion about the effect of AC driving frequency on the SPP modes generated at the junction area and also at the end of plasmonic waveguides. Figure S1 shows the real-plane images of light emission collected from tunnel junctions, where Cu was grounded and Al was biased. To analyze the excited and propagated SPP modes, we have installed a pinhole along the collection path to select areas of interest and collect light emission at the junction center (upward dotted circle) and the end of waveguides (downward dotted circle). As is shown in Figure S1, light emission was observed mainly from the junction area and the Cu waveguiding edge, which agrees well with our previous result. Figure S2 and Figure S3 illustrate the back focal-plane images of light emission collected from the center of tunnel junctions and the waveguiding edge, respectively. The inner and outer arcs correspond to the scattering of Cu-air (N.A = 1.0) and Cu-SiO_2_ (N.A = 1.49) SPP modes. We observe that the excited SPP modes at the junction area and the waveguiding edge do not change at different AC driving frequencies, even though the corresponding light-emitting intensity is significantly different.


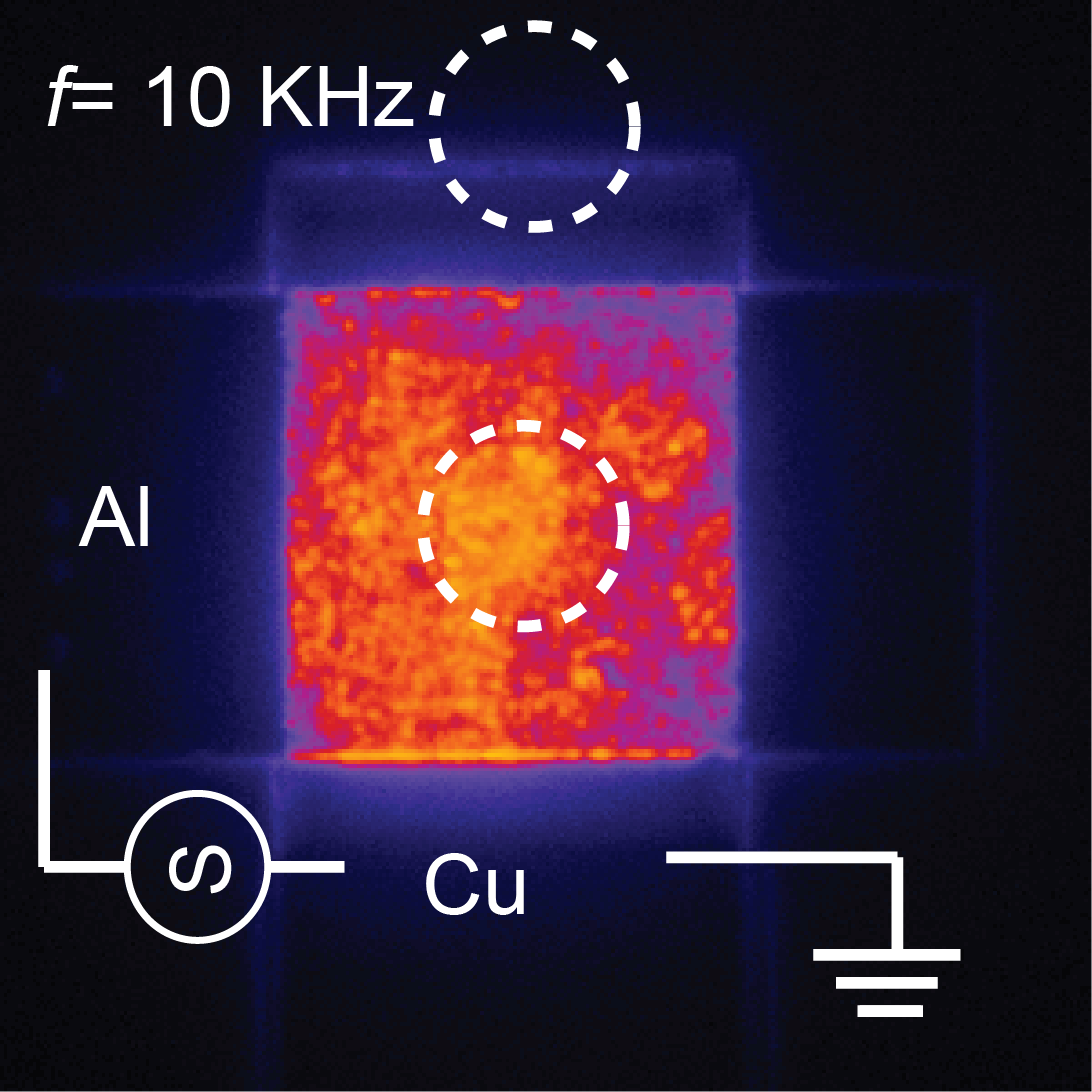


**Figure S1.** Real-plane images of light emission collected from Al/AlO_X_/Cu tunnel junctions. Cu was grounded, and Al was biased negatively for larger breakdown voltages. The two dotted circles represent the collection area of the junction center and waveguide end shown in Figures S2 and S3, receptively.


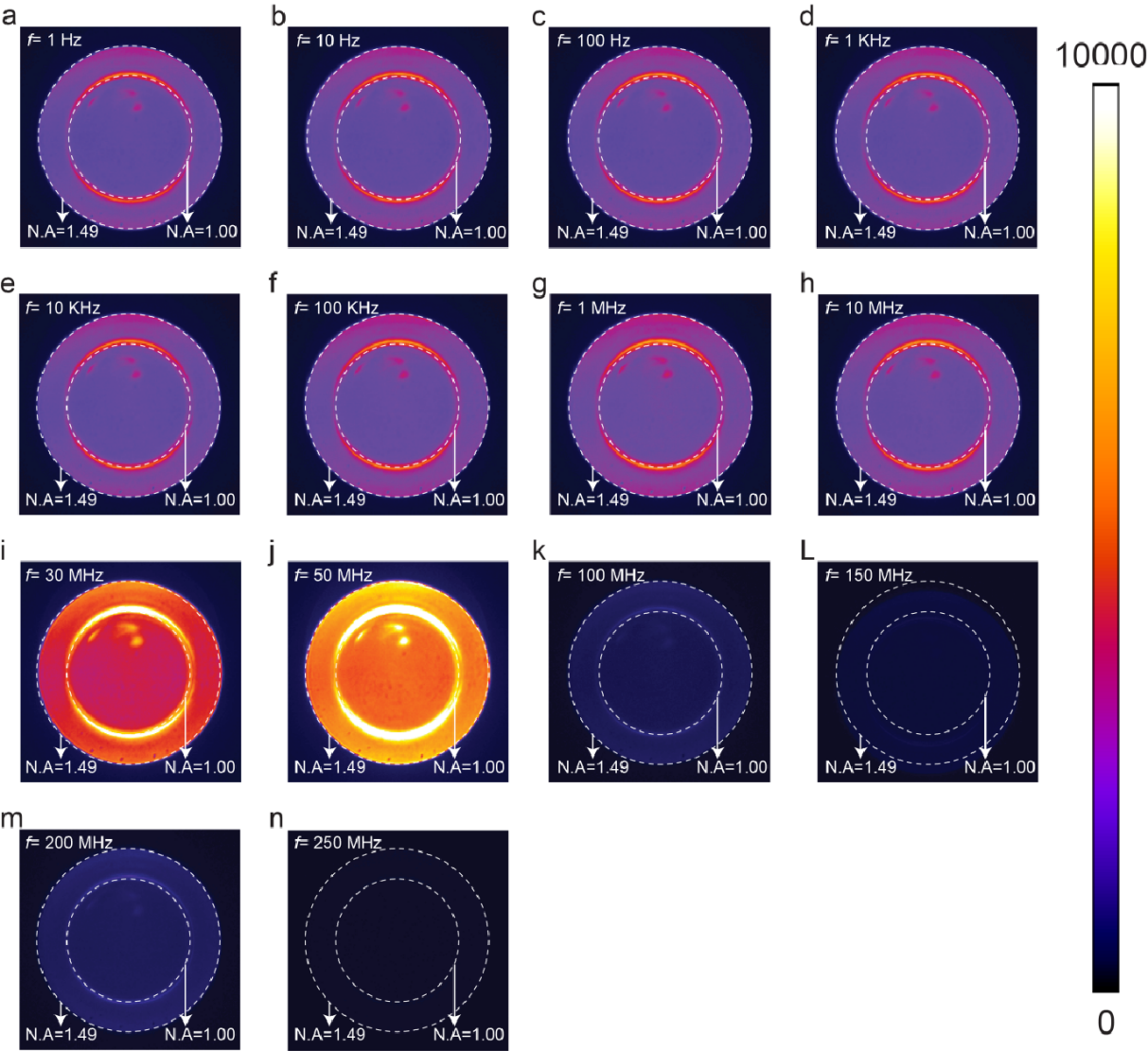


**Figure S2.** Frequency-dependent back focal-plane images of light emission collected only from the center of tunnel junctions (downward circle area in Figure S1). The inner and outer arcs correspond to the scattering of Cu-air and Cu-SiO_2_ SPP modes.


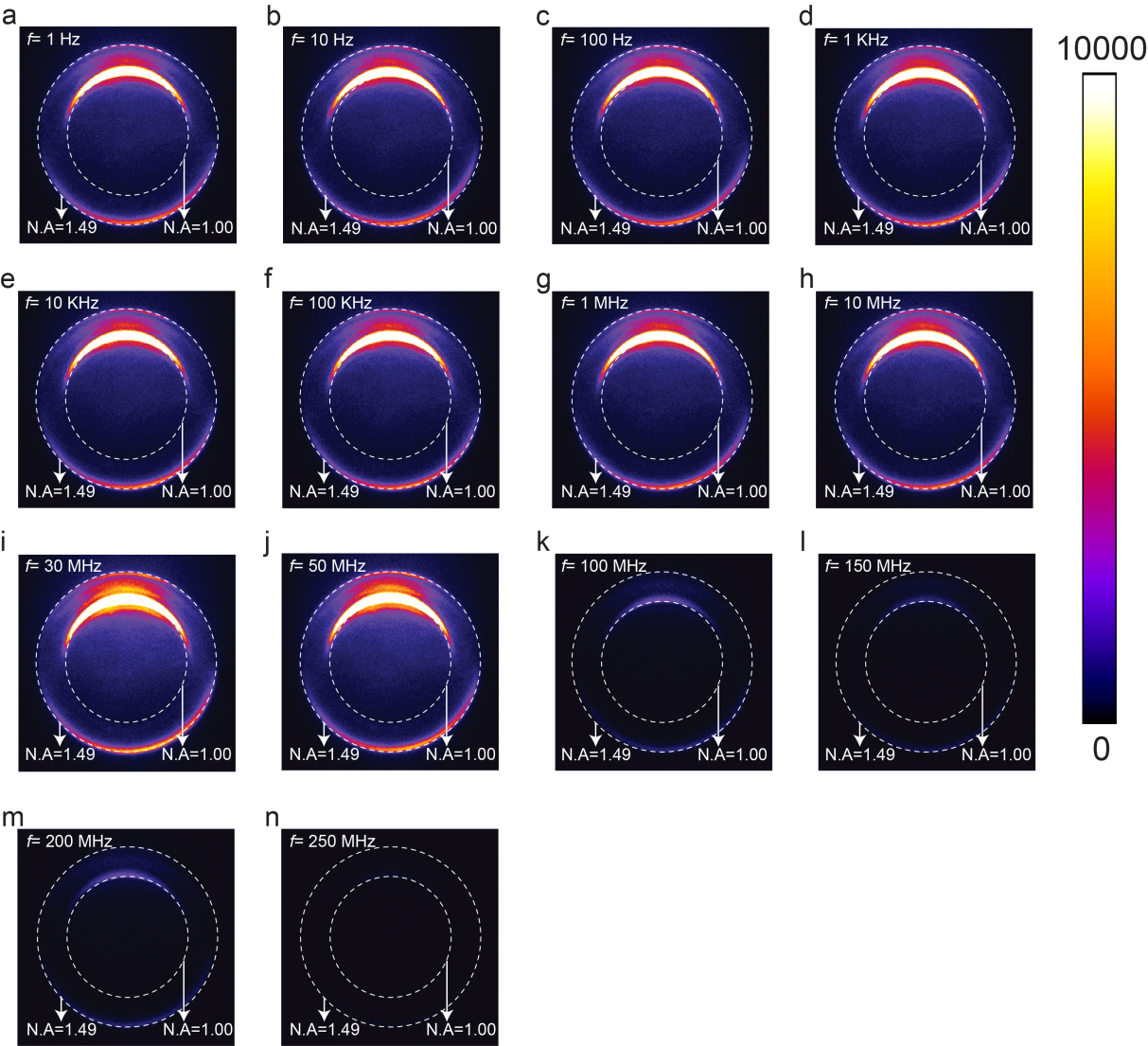


**Figure S3.** Frequency-dependent back-plane images of light emission collected only from the end of Cu waveguides (upward circle area in Figure S1).

## S3. Cutoff frequency extraction and RC constant of tunnel junctions

In principle, the ultimate modulation speed of our demonstrated electronic-plasmonic modulators is limited by the tunneling time of electrons through the barriers at a frequency of a few THz.^[6-8]^ However, experimental verification of electronic-plasmonic modulation at the THz regime is hindered by the RC time constant of the junction itself and the impedance matching of electric connections. Thus, it is vital to measure the RC constant of tunnel junctions and extract the corresponding cutoff frequency. An R&S FSV40 VNA was used to measure the one-port S-parameter of the junctions. GSG probes were attached to the input pads of a junction. A probe calibration was performed to shift the reference plane to the tip of the probe. Figure S4a shows the equivalent circuit model diagram of tunnel junctions, where *L*_S_, *R*_S_, *C*_j_, and *R*_j_ are the series inductance, connection resistance, capacitance, and resistance of the junction, respectively. Figure S4b illustrates the measured and fitted impedance plotted against the applied RF frequency and the corresponding cutoff frequency is fitted to be 8.5 GHz, which is 1-2 orders of magnitude higher than the applied maximum RF frequency when we measure modulation of light emission and plasmonic signals.


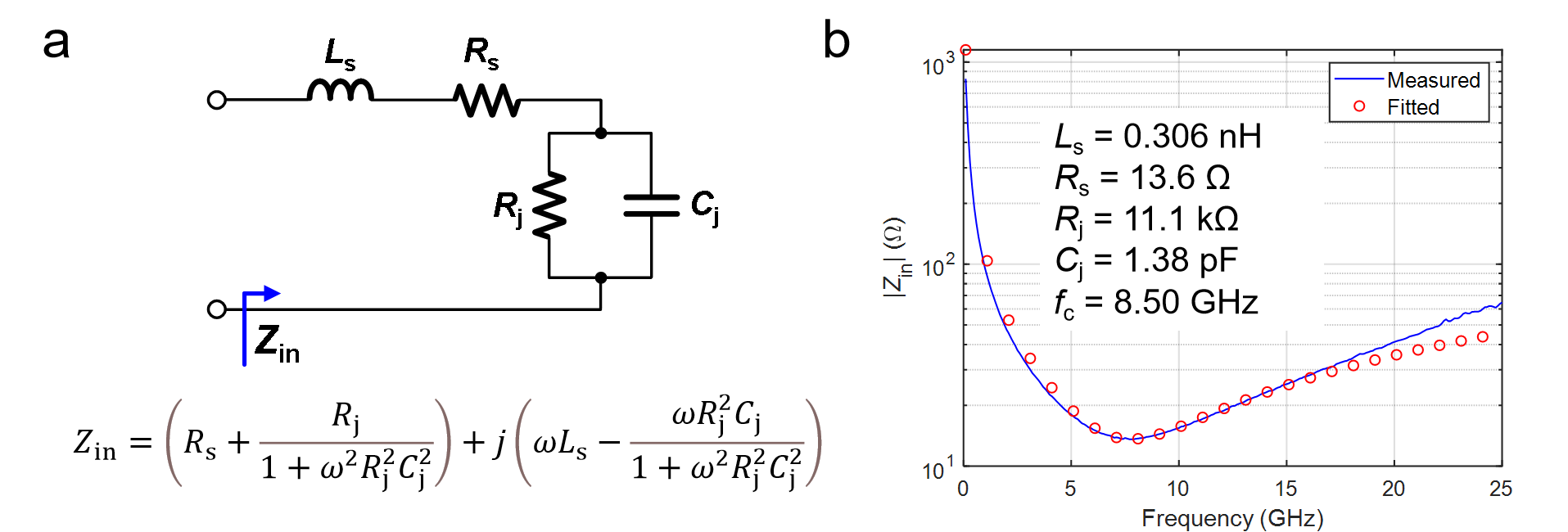


**Figure S4.** Estimation of the cut-off frequency. a) Equivalent circuit model diagram of tunnel junctions. *L*_S_, *R*_S_, *C*_j_, and *R*_j_ are the series inductance, connection resistance, capacitance, and resistance of the junction, respectively. The fitted equation of the equivalent circuit model is also presented below the diagram. b) The measured and fitted impedance plotted against the applied RF frequency. The tunnel junction area is 5×5 μm^2^.

## S4. Frequency-dependent reflection coefficient at the input of tunnel junctions.

An R&S FSV40 VNA was used to measure the Reflection coefficient at the input of the junction when the applied frequency ranges from 10 MHz to 1 GHz. Figure S5a presents the equivalent circuit model diagram of a lossless matching network, where *Γ*(*ω*) is defined as the reflection coefficient at the input of the junction. Figure S5b shows the plot of the reflection coefficient *Γ*(*ω*) as a function of input frequency. We observe that there is a dip in the reflection coefficient (from the measured junction impedance) between 10 and 100 MHz, suggesting the power delivered from the power source to the junction has a peak within this frequency range. This is the reason why the measured modulated current (Figure 3d), as well as light-emitting intensity (Figure 4c) increases when the applied frequency increases beyond 10 MHz and finally decreases shapely beyond 100 MHz. Due to the Bode-Fano criterion,^[9-10]^ broadband matching for parallel RC load impedance is difficult to implement experimentally as shown in our demonstration. Therefore, in future work, there are two possible ways to overcome the impedance mismatch: 1) narrow bandwidth (single frequency) matching, which may complicate the whole measurement; 2) using a high RF power source (signal generator and amplifier) to let sufficient power reach the tunnel junctions.^[8, 11]^


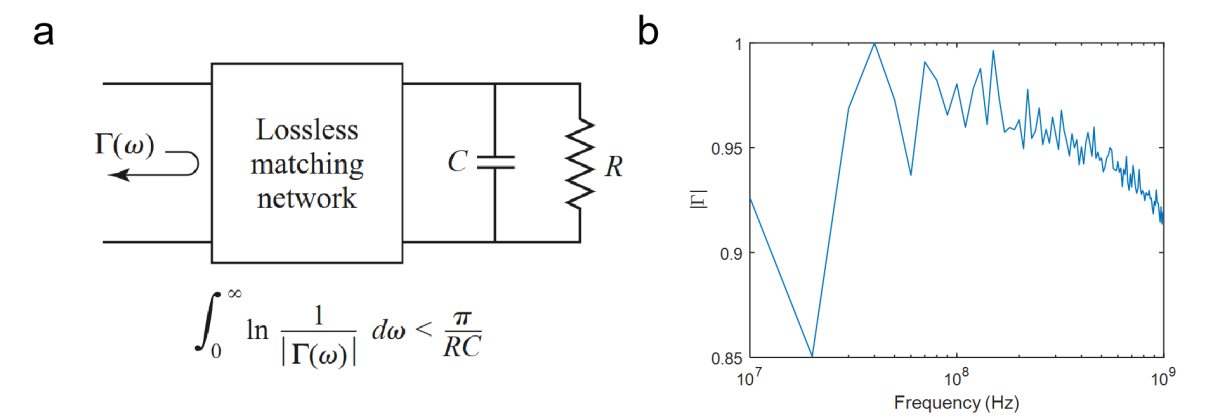


**Figure S5.** Reflection coefficient at the input of the junction. a) Equivalent circuit model diagram of a lossless matching network and the Bode-Fano Criterion equation. b) Frequency-dependent reflection coefficient at the input of the junction.

**Reference**

1. Du, W., et al., *Highly efficient on-chip direct electronic–plasmonic transducers.* Nature Photonics, 2017. **11**(10): p. 623-627.

2. Parzefall, M., et al., *Antenna-coupled photon emission from hexagonal boron nitride tunnel junctions.* Nature Nanotechnology, 2015. **10**(12): p. 1058-1063.

3. Wang, F., et al., *Spatial Control over Stable Light-Emission from AC-Driven CMOS-Compatible Quantum Mechanical Tunnel Junctions.* Laser & Photonics Reviews, 2022. **16**(5): p. 2100419.

4. Sadaf, S. M., et al., *Alternating-Current InGaN/GaN Tunnel Junction Nanowire White-Light Emitting Diodes*. Nano Letters, 2015. **15**(10): p. 6696-6701.

5. Wang, F., et al., *CMOS-Compatible Electronic–Plasmonic Transducers Based on Plasmonic Tunnel Junctions and Schottky Diodes.* Small, 2022. **18**(1): p. 2105684.

6. Février, P. and J. Gabelli, *Tunneling time probed by quantum shot noise.* Nature Communications, 2018. **9**(1): p. 4940.

7. Parzefall, M., et al., *Light from van der Waals quantum tunneling devices.* Nature Communications, 2019. **10**(1): p. 292.

8. Parzefall, M. and L. Novotny, *Light at the End of the Tunnel.* ACS Photonics, 2018. **5**(11): p. 4195-4202.

9. Yang, X., et al., *Broadband Time-Modulated Absorber beyond the Bode-Fano Limit for Short Pulses by Energy Trapping.* Physical Review Applied, 2022. **17**(4): p. 044003.

10. Shlivinski, A. and Y. Hadad, *Beyond the Bode-Fano Bound: Wideband Impedance Matching for Short Pulses Using Temporal Switching of Transmission-Line Parameters.* Physical Review Letters, 2018. **121**(20): p. 204301.

11. Hervé, M., et al., *High frequency transmission to a junction of a scanning tunneling microscope.* Applied Physics Letters, 2015. **107**(9).
